# Mechanism of Action of an Environmentally Relevant Organochlorine Mixture in Repressing Steroid Hormone Biosynthesis in Leydig Cells †

**DOI:** 10.3390/ijms23073997

**Published:** 2022-04-03

**Authors:** Annick N. Enangue Njembele, Zoheir B. Demmouche, Janice L. Bailey, Jacques J. Tremblay

**Affiliations:** 1 Reproduction, Mother and Child Health, Room T3-67, Centre de Recherche du CHU de Québec–Université Laval, CHUL, 2705 Laurier Blvd., Quebec City, QC G1V 4G2, Canada; enangue7@yahoo.fr (A.N.E.N.); zoheir.demmouche.1@ulaval.ca (Z.B.D.); 2 Centre for Research in Reproduction, Development and Intergenerational Health, Department of Animal Science, Faculty of Agricultural and Food Sciences, Université Laval, Quebec City, QC G1V 0A6, Canada; janice.bailey@frq.gouv.qc.ca; 3 Centre for Research in Reproduction, Development and Intergenerational Health, Department of Obstetrics, Gynecology, and Reproduction, Faculty of Medicine, Université Laval, Quebec City, QC G1V 0A6, Canada

**Keywords:** testis, Leydig cells, steroidogenesis, STAR, environmental toxicology, endocrine disrupters, organochlorine, proteomics

## Abstract

Within Leydig cells, steroidogenesis is induced by the pituitary luteinizing hormone (LH). The binding of LH to its receptor increases cAMP production, which then activates the expression of genes involved in testosterone biosynthesis. One of these genes codes for the steroidogenic acute regulatory (STAR) protein. STAR is part of a complex that shuttles cholesterol, the precursor of all steroid hormones, through the mitochondrial membrane where steroidogenesis is initiated. Organochlorine chemicals (OCs) are environmental persistent organic pollutants that are found at high concentrations in Arctic areas. OCs are known to affect male reproductive health by decreasing semen quality in different species, including humans. We previously showed that an environmentally relevant mixture of OCs found in Northern Quebec disrupts steroidogenesis by decreasing STAR protein levels without affecting the transcription of the gene. We hypothesized that OCs might affect STAR protein stability. To test this, MA-10 Leydig cell lines were incubated for 6 h with vehicle or the OCs mixture in the presence or absence of 8Br-cAMP with or without MG132, an inhibitor of protein degradation. We found that MG132 prevented the OC-mediated decrease in STAR protein levels following 8Br-cAMP stimulation. However, progesterone production was still decreased by the OC mixture, even in the presence of MG132. This suggested that proteins involved in steroid hormone production in addition to STAR are also affected by the OC mixture. To identify these proteins, a whole cell approach was used and total proteins from MA-10 Leydig cells exposed to the OC mixture with or without stimulation with 8Br-cAMP were analyzed by 2D SDS-PAGE and LC-MS/MS. Bioinformatics analyses revealed that several proteins involved in numerous biological processes are affected by the OC mixture, including proteins involved in mitochondrial transport, lipid metabolism, and steroidogenesis.

## 1. Introduction

Mammalian reproductive function is controlled by various hormones produced by endocrine glands that compose the hypothalamic-pituitary-gonadal axis. Leydig cells are located in the male gonad and are the main producers of testosterone. Testosterone is responsible for male sex differentiation during fetal life, puberty (reviewed in [1]), acquisition of secondary sexual characteristics (reviewed in [1]), and initiation and maintenance of spermatogenesis (reviewed in [2,3]).

Leydig cell steroidogenesis starts with the transport of cholesterol—the substrate for the biosynthesis of all steroid hormones—through the inner mitochondrial membrane. This transport step is essential because the CYP11A1 enzyme responsible for the first reaction where cholesterol is transformed into pregnenolone is located on the matrix side of the inner mitochondrial membrane (reviewed in [4]). Pregnenolone then diffuses and enters the endoplasmic reticulum where three remaining enzymes (CYP17A1, HSD3B1, and HSD17B3) will act sequentially to transform pregnenolone into testosterone (reviewed in [4]). Cholesterol shuttling through mitochondrial membranes is considered the rate-limiting step of steroidogenesis. This step is carried out by the transduceosome, a multiprotein complex containing the steroidogenic acute regulatory protein (STAR) (reviewed in [5,6,7,8]). Leydig cells express the luteinizing hormone (LH) receptor (LHGCR), and stimulation with LH leads to the activation of several signaling pathways and transcription factors downstream of LHCGR, ultimately resulting in an increase in *Star* gene transcription (reviewed in [9,10]). Insufficient STAR expression and/or action in humans has been shown to cause congenital adrenal hyperplasia associated with male ambiguous genitalia due to a significant reduction in adrenal and testicular steroidogenesis ([11] and reviewed in [12]). A similar phenotype is observed in *Star*-deficient mice [13,14].

It is widely recognized that endocrine-disrupting chemicals affect Leydig cell function in several species, including humans, resulting in hormone insufficiency and incomplete male sex differentiation (reviewed in [15]). There is a growing body of evidence showing that a reduction in *Star* gene expression leading to decreased steroidogenesis is a result of exposure to several endocrine disrupting chemicals (reviewed in [16]), including phthalates [17] and organochlorines [18,19]. Organochlorines are a family of organic compounds containing at least one covalently bonded chlorine atom. Lindane, an organochlorine pesticide, also called gamma-hexachlorocyclohexane (γ-HCH), inhibits steroidogenesis by decreasing STAR protein levels in the MA-10 mouse Leydig cell line [19]. In addition, it has been reported that the synthetic organochlorine methoxychlor (or its metabolites), a derivative of DDT, affects Leydig cell steroidogenesis by decreasing the expression and activity of CYP11A1 [20,21].

We have previously shown that an environmentally relevant organochlorine mixture similar to that present in Northern Quebec disrupts mouse Leydig cell steroidogenesis by decreasing STAR protein levels without affecting *Star* gene transcription [18]. However, the mechanism of action whereby organochlorines cause a reduction in STAR protein levels remains unknown. Here we report that the organochlorine mixture acts by increasing STAR protein degradation. In addition, we have used a large-scale proteomics approach and identified several additional proteins likely affected by the organochlorine mixture in Leydig cells, some of which were validated by Western blot.

## 2. Results

### 2.1. The OC Mixture Affects STAR Protein Degradation

The OC mixture is composed of over 15 OCs found in the diet of the Inuit population of Northern Québec (Table 1). Exposure of Leydig cell lines to this OC mixture decreases STAR protein levels without affecting *Star* gene expression [18]. We therefore surmised that the OC mixture might interfere with STAR protein synthesis and/or degradation.

**Figure 1:**
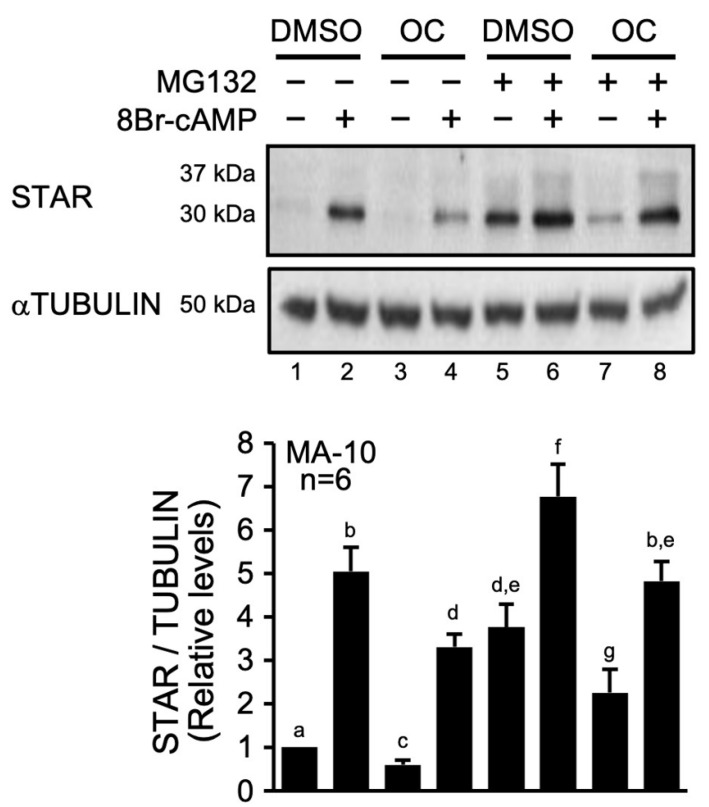
The OC mixture affects STAR protein levels in MA-10 Leydig cells by acting on the protein degradation pathway. MA-10 Leydig cells were treated with either dimethylsulfoxyde (DMSO) or 10 µg/mL of an environmentally relevant mixture of OCs for 2 h and then treated for another 4 h with (+) or without (−) of 0.1 mM 8Br-cAMP and 10 µM MG132 (an inhibitor of protein degradation) as indicated, and STAR protein was detected by Western blot. αTUBULIN was used as a loading control. The experiment was repeated six times, and the results from each experiment were quantified and plotted as mean ± SE of the mean. Different letters indicate a statistically significant difference between groups (*p* < 0.05).

To test whether the OC mixture affects STAR protein stability, MA-10 Leydig cells were treated with the OC mixture with or without 8Br-cAMP in the presence or absence of MG132 (10 µM), an inhibitor of the proteasomal degradation pathway, and to a certain extent, the mitochondrial matrix protein degradation pathway [23,24]. As expected, STAR protein levels were induced approximately 5-fold following 8Br-cAMP stimulation (Figure 1, lanes 1 and 2). Although the response to 8Br-cAMP was maintained in the presence of the OC mixture (Figure 1, lanes 3 and 4), overall STAR protein levels were significantly reduced (compare lane 4 with lane 2 in Figure 1). When MG132 was added, an accumulation of the 30 kDa STAR protein was observed, both in unstimulated (compare lane 5 with lane 1 in Figure 1) and in 8Br-cAMP-stimulated (compare lane 6 with lane 2 in Figure 1) MA-10 Leydig cells. This increase in the level of the 30 kDa form in the presence of MG132 was still considerably reduced after exposure to the OC mixture (compare lanes 7 and 5 in Figure 1), indicating that the OC mixture continues to affect the accumulation/stabilization of 30 kDa STAR induced by MG132. Furthermore, the presence of MG132 caused an increase in the 37 kDa cytoplasmic STAR preprotein form, which is more easily detected after 8Br-cAMP treatment (Figure 1, lanes 6 and 8), consistent with a previous study [24]. Altogether, these data indicate that the OC mixture reduces hormone-induced steroidogenesis by decreasing the level of the mature 30 kDa STAR protein within the mitochondrial matrix by a mechanism involving protein degradation.

### 2.2. Inhibition of Protein Degradation and the OC Mixture Both Repress Progesterone Synthesis

**Figure 2:**
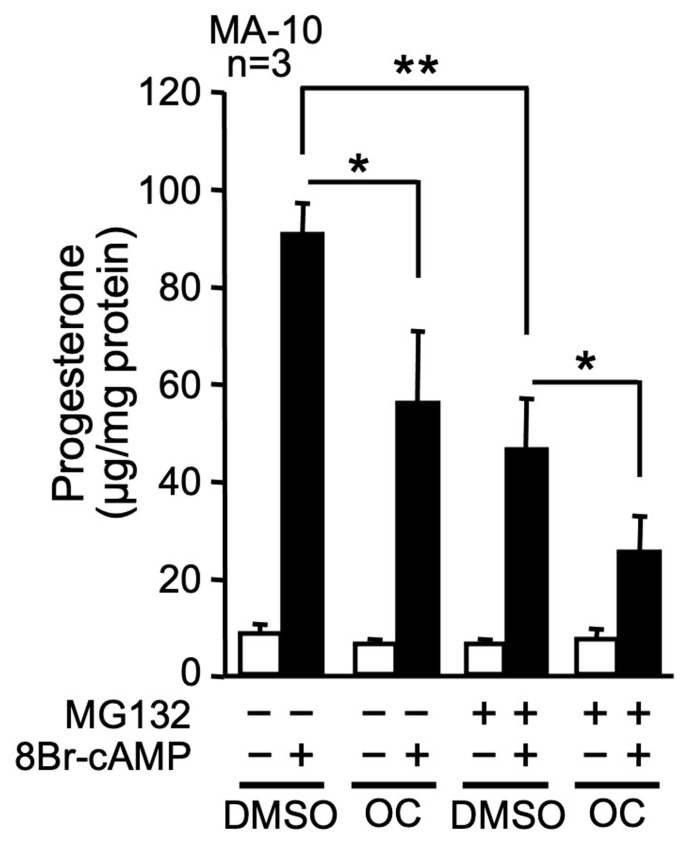
The OC mixture affects steroidogenesis, even when protein degradation is inhibited. MA-10 Leydig cells were treated with either DMSO or 10 µg/mL of OC mixture for 2 h and then treated for another 4 h with (+) or without (−) 0.1 mM 8Br-cAMP and 10 µM MG132 (an inhibitor of protein degradation) as indicated. Progesterone production was quantified by ELISA and corrected according to protein concentration. Values are the mean of three individual experiments, each performed in duplicate (±SEM). *: *p* < 0.05, **: *p* < 0.01.

Next, we evaluated progesterone production after exposure to the OC mixture in the presence or absence of the proteasome inhibitor MG132. The OC mixture did not significantly affect basal progesterone production, while 8Br-cAMP-induced progesterone production was reduced by about 40% (Figure 2). In the presence of MG132 to inhibit protein degradation, the 8Br-cAMP-mediated increase in progesterone production was significantly reduced from 10.2- to 7.7-fold (Figure 2). When the OC mixture and MG132 were combined, 8Br-cAMP-induced progesterone production was more potently reduced to 3.5-fold compared to 10.2-fold for the control (Figure 2). These results indicate that proper protein degradation is essential for maximal hormone-induced steroid hormone production and that proteins other than STAR are likely targeted by the OC mixture in MA-10 Leydig cells.

### 2.3. Exposure to the OC Mixture Affects Several Proteins and Biological Processes in Leydig Cells

**Figure 3:**
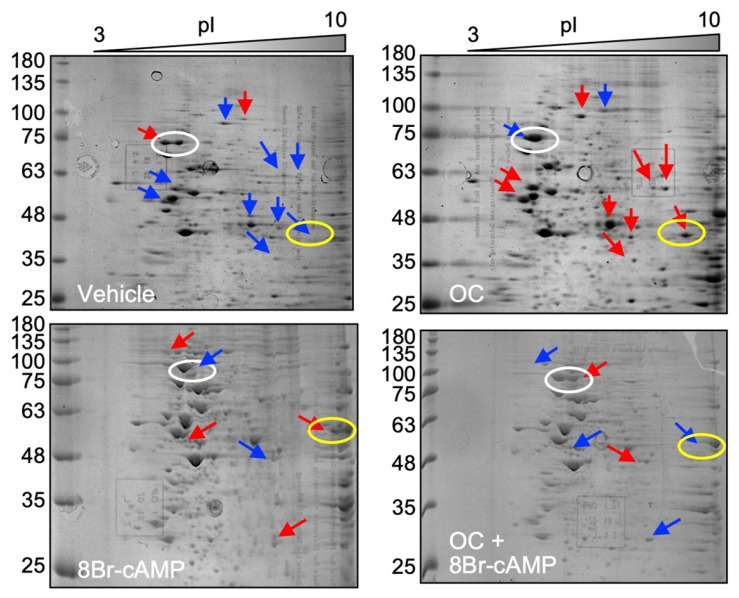
Exposure to the OC mixture affects several proteins in Leydig cells. MA-10 Leydig cells were treated with either DMSO or 10 µg/mL of the OC mixture for 2 h and then treated for another 4 h with or without 0.1 mM 8Br-cAMP as indicated. The Coomassie blue-stained two-dimensional SDS-PAGE gels were scanned, and spots were matched and quantified using ImageMaster 2D Platinum 6.0 software. Red and blue arrows represent increased and decreased protein spots, respectively. Circled spots (white = Sample 1; yellow = Sample 2) were analyzed by LC-MS/MS for protein identification.

To identify other potential targets of the OC mixture, whole cell extracts were isolated from MA-10 Leydig cells treated with the OC mixture in the presence or absence of 8Br-cAMP and analyzed on a two-dimensional (isoelectric point and molecular weight) SDS-PAGE followed by Coomassie blue staining. The gels were scanned, and images were analyzed using ImageMaster 2D Platinum 6.0 software (GE Healthcare Life Sciences, Baie d’Urfé, QC, Canada), allowing gel matching and spot quantification. As shown in Figure 3, several spots were differentially modulated when comparing each treatment condition; increased spots are represented by red arrows and decreased spots by blue arrows. A sampling of spots that were different between the various conditions were excised from the gel for each treatment and analysed by LC-MS/MS. As shown in Figure 3, the white circles correspond to Sample 1 and the yellow circles to Sample 2. A total of 37 proteins were present in the four conditions of Sample 1 and 66 in the four conditions of Sample 2. A detailed bioinformatic analysis of the proteins present in each sample was then performed.

**Figure 4:**
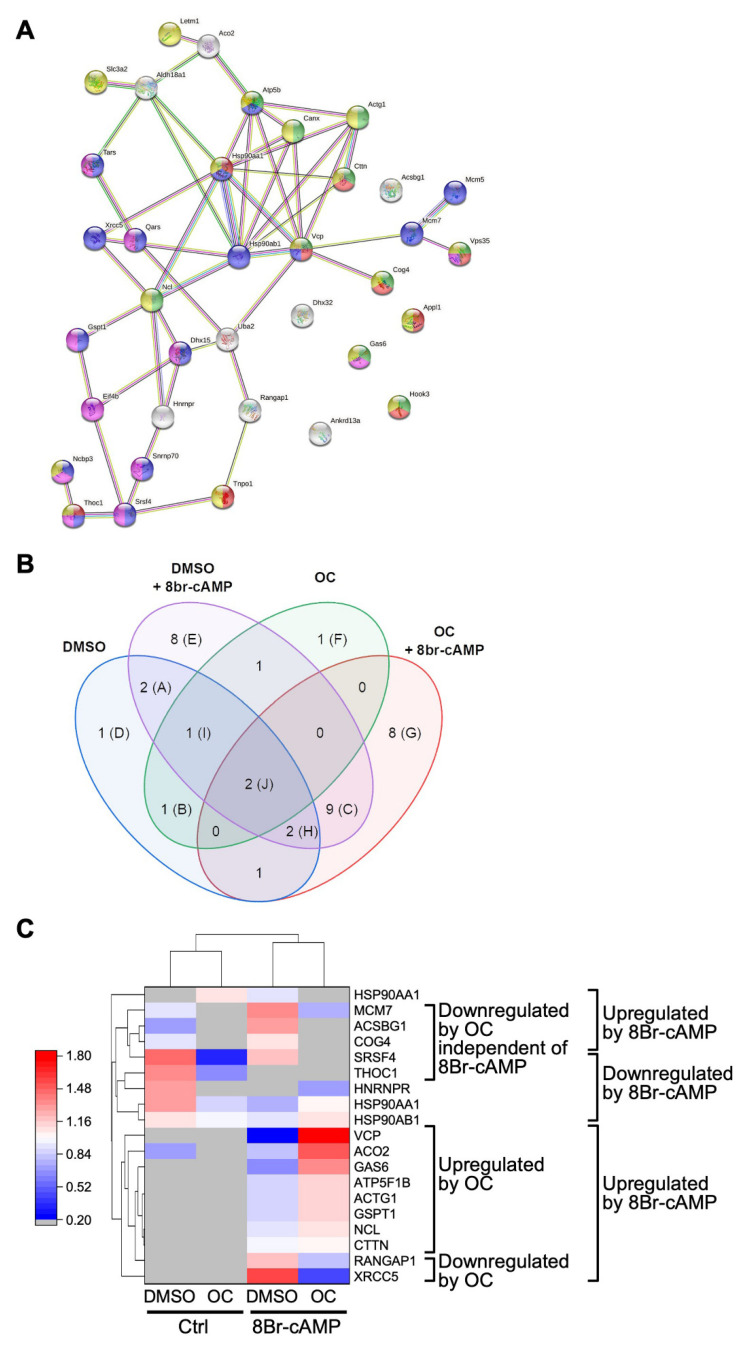
Proteomic profiling of Sample 1 shows proteins that are potentially affected after exposure of Leydig cells to an OC mixture. MA-10 Leydig cells were treated with either DMSO or 10 µg/mL of the OC mixture for 2 h and then treated for another 4 h with or without 0.1 mM 8Br-cAMP, as indicated. Protein content in the spots corresponding to Sample 1 (white circles in Figure 3) was determined by LC-MS/MS. Results were then analyzed using Scaffold software with protein threshold 99%, minimum unique peptide 2 and peptide threshold 95%. (**A**) The proteins present in Sample 1 were further analyzed using the String software to identify interactions and associations between the various proteins and their roles in biological processes (listed in Table 2). The lines between the nodes correspond to the type/strength of an interaction according to annotations in String. The colors of the nodes refer to the following biological processes: green = vesicle-mediated transport, red = protein transport, purple = nucleobase-containing compound metabolic process, pink = gene expression, yellow = transport. (**B**) The OriginPro Version 2021 software was used to generate a Venn diagram identifying the protein present in each condition, as indicated. The proteins are listed in Table 3. (**C**) Potentially differentially regulated proteins in each condition (as indicated) are shown as a heatmap generated using the OriginPro Version 2021 software. The protein names and whether they are up/downregulated after a treatment are indicated on the right of the heatmap. A relative intensity scale is shown on the left. Proteins that were below the detection level are represented by a gray rectangle.

The proteins in Sample 1 (white circles in Figure 3) were first analyzed qualitatively using the String software to identify functional protein association and interaction networks. As shown in Figure 4A, this analysis revealed that several of the proteins are known to act in common biological processes. These processes are listed in Table 2 and include proteins involved in protein transport, metabolic process, and gene expression.

Next, a Venn diagram was created to highlight the differences in the protein content between the four experimental conditions (DMSO, DMSO+OC, 8Br-cAMP, 8Br-cAMP+OC) for Sample 1. As shown in Figure 4B, protein content varied significantly between the four conditions. For instance, eight proteins were unique to the 8Br-cAMP+OC condition (identified by the letter G in Figure 4B and listed in column G of Table 3). On the other hand, other proteins were common to several conditions. For instance, two proteins were present in all four conditions (identified by the letter J in Figure 4B and in column J of Table 3).

Although the data presented in the Venn diagram indicate whether a protein is present or absent from the sample analyzed, it does not provide any information regarding protein level. This was determined by comparing the relative abundance of a given protein detected in each of the four treatment conditions. The data are presented as a heatmap shown in Figure 4C. Although the stimulation with 8Br-cAMP increased the level of several proteins as expected, treatment with the OC mixture potentially affected the level of the various proteins. As shown in Figure 4C, seven proteins appeared downregulated in response to OC exposure, while eight appeared upregulated. The potentially downregulated proteins include proteins involved in DNA repair (XRCC5) and fatty acid metabolism (ACSBG1). Potentially upregulated proteins included GSPT1 involved in translation termination, GAS6 involved in growth arrest, and VCP that regulates ER stress. The main functions (Uniprot knowledge database, accessed on 6 December 2021, www.uniprot.org) of the proteins from Sample 1 up/downregulated after exposure to the OC mixture are listed in Table 4.

**Figure 5:**
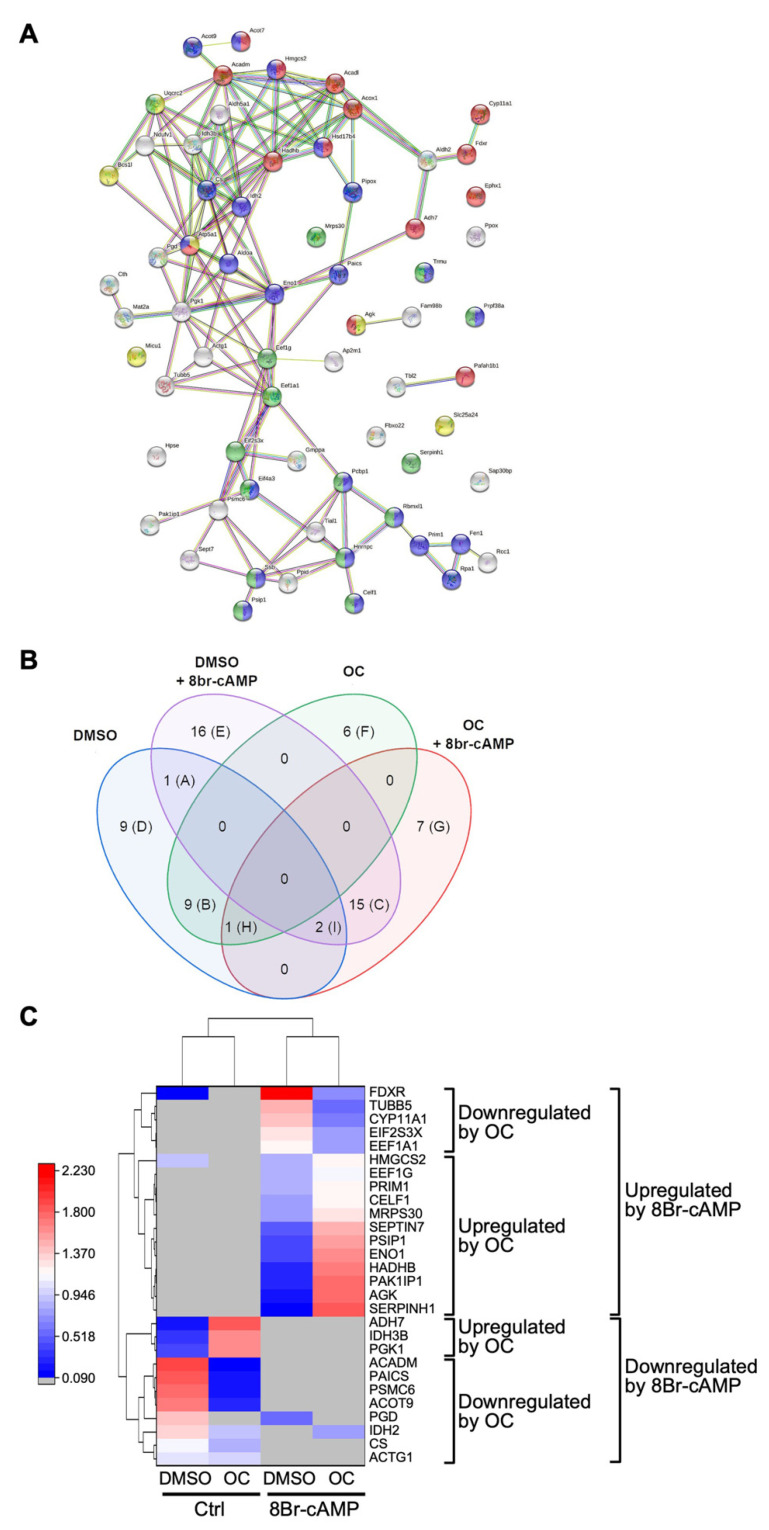
Proteomics profiling of Sample 2 shows proteins that are potentially affected after exposure of Leydig cells to an OC mixture. MA-10 Leydig cells were treated with either DMSO or 10 µg/mL of the OC mixture for 2 h and then treated for another 4 h with or without 0.1 mM 8Br-cAMP as indicated. Protein content in the spots corresponding to Sample 2 (yellow circles in Figure 3) was determined by LC-MS/MS. Results were then analyzed using Scaffold software with protein threshold 99%, minimum unique peptide 2 and peptide threshold 95%. (**A**) The proteins present in Sample 2 were further analyzed using the String software to identify the interactions and associations between the various proteins and their implications in biological processes (listed in Table 4). The lines between the nodes correspond to the type/strength of an interaction according to annotations in String. The colors of the nodes refer to the following biological processes: green = gene expression, red = lipid metabolic process, purple = nucleobase-containing compound metabolic process, yellow = mitochondrial transport. (**B**) OriginPro Version 2021 software was used to generate a Venn diagram identifying the protein present in each condition, as indicated. The proteins are listed in Table 5. (**C**) Putative differentially regulated proteins in each condition (as indicated) are shown as a heatmap generated using the OriginPro Version 2021 software. The protein names and whether they are up/downregulated after a treatment are indicated on the right of the heatmap. A relative intensity scale is shown on the left. Proteins that were below the detection level are represented by a gray rectangle.

A similar approach was used to study the proteins present in Sample 2 (yellow circles in Figure 3). As shown in Figure 5A, the String analysis to identify functional protein association and interaction networks revealed clusters of proteins regulating common biological processes. These biological processes are listed in Table 5 and include mitochondrial transport as well as the lipid metabolic process, both of which are important for Leydig cell function.

Qualitative analysis (presence vs. absence) of the proteins in the four treatment conditions for Sample 2 also revealed potentially significant differences, as presented in the Venn diagram of Figure 5B. For instance, 6 proteins are only found in the OC mixture-treated conditions in the absence of 8Br-cAMP (identified by the letter F in Figure 5B and listed in column F of Table 6). In the presence of 8Br-cAMP, 16 proteins are identified (letter E in Figure 5B and column E in Table 6) while in the 8Br-cAMP+OC condition, 7 unique proteins are found (letter G in Figure 5B and column G in Table 6). 

Finally, a heatmap was also generated to identify the potential changes in protein levels between the four treatment conditions for Sample 2. As shown in Figure 5C, 8Br-cAMP treatment led to increased levels of several proteins, including proteins known to be involved in steroidogenesis such as FDXR and CYP11A1. Exposure to the OC mixture appeared to modulate the levels of several proteins; 8 were downregulated and 15 upregulated. Potentially downregulated proteins included proteins involved in steroidogenesis (FDXR, CYP11A1), protein synthesis (EIF2S3X, EEF1A1), lipid and carbohydrate metabolism (ACADM, ACOT9, PGD), and energy production (IDH2, CS). Potentially upregulated proteins were associated with cholesterol synthesis (HMGCS2), DNA synthesis (PRIM1), RNA splicing (CELF1), lipid metabolism (HADHB, AGK), and retinoid metabolism (ADH7). The main function (Uniprot knowledge database, accessed on 6 December 2021, www.uniprot.org) of the proteins from Sample 2 up/downregulated after exposure to the OC mixture is listed in Table 7.

**Figure 6:**
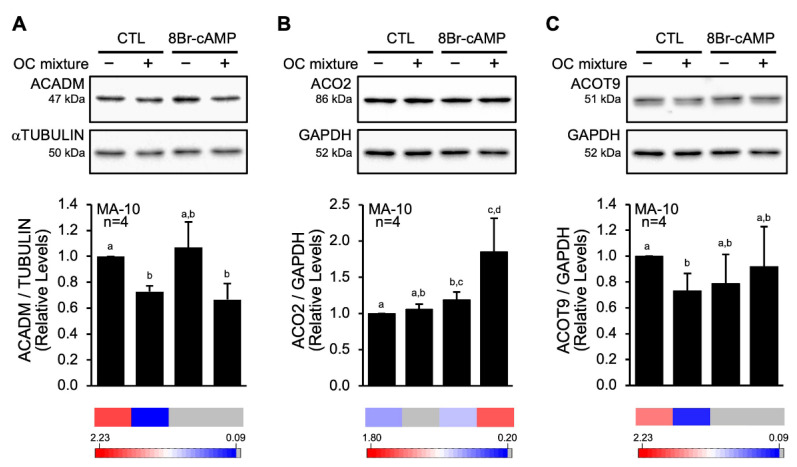
The OC mixture affects protein levels in MA-10 Leydig cells. MA-10 Leydig cells were treated with either dimethylsulfoxyde (−) or 10 µg/mL of an environmentally relevant mixture of OCs (+) for 2 h and then treated for another 4 h without (CTL) or with 0.1 mM 8Br-cAMP as indicated. ACADM (**A**), ACO2 (**B**), and ACOT9 (**C**) proteins were detected by Western blot. αTUBULIN or GAPDH were used as loading controls. The experiment was repeated four times, and the results from each experiment were quantified and are plotted as mean ± SE of the mean. Different letters indicate a statistically significant difference between groups (*p* < 0.05). The relevant line from the heatmap analysis (Figure 4 and Figure 5) is shown under the graph.

Although bioinformatics analyses of the data obtained by mass spectrometry suggest that the level of several proteins is likely affected following exposure to the OC mixture, it is important to validate a snapshot of the proteins affected by Western blot. We therefore treated MA-10 Leydig cells with the same conditions as described above (DMSO, 8Br-cAMP, OC mixture, 8Br-cAMP+OC mixture), and total proteins were isolated and Western blots were performed for three proteins identified as potentially deregulated: ACADM, ACO2, and ACOT9. As shown in Figure 6A, the levels of ACADM, a protein involved in fatty acid metabolism, are reduced following exposure to the OC mixture. The levels of another protein analyzed, ACO2, a protein involved in the Krebs cycle, were not affected by either 8Br-cAMP or the OC mixture individually but were increased in the presence of both 8Br-cAMP+OC mixtures (Figure 6B). The last protein to be validated was ACOT9, an enzyme involved in fatty acid metabolism. As shown in Figure 6C, ACOT9 levels were reduced following exposure to the OC mixture.

## 3. Discussion

The aim of this study was to provide a better understanding of the mechanisms of action of an environmentally relevant OC mixture in inhibiting steroidogenesis in Leydig cells.

### 3.1. The OC Mixture Modulates STAR Protein Processing

Although the exact mechanism by which STAR contributes to the transport of cholesterol to the inner mitochondrial membrane remains to be fully elucidated, it is accepted that STAR processing and translocation into the mitochondria is a regulated process and decreased or increased STAR processing has significant consequences for the rate of steroidogenesis (reviewed in [25]).

The STAR protein is produced as a cytoplasmic 37 kDa precursor protein, and upon its import into the mitochondrial matrix, the 37 kDa precursor protein is processed by mitochondrial peptidases generating the mature 30 kDa form [26,27,28]. Processing of the STAR protein in Leydig cells is highly regulated and involves the proteasome as well as proteases found in the mitochondrial matrix [24,29]. The active form of STAR involved in the transport of cholesterol through the mitochondrial membrane is the 37 kDa precursor [30]. However, it is also known that the 37 kDa form has a very short half-life of about 5 min in the cytosol, based on the processing rate of the signal sequence [30,31]. The proteasome plays an important role in degrading the non-imported 37 kDa precursor protein [29,32]. On the other hand, the 30 kDa form located in the mitochondrial matrix has a longer half-life of about 4–5 h [23,24]. This explains why, in most studies, including our own, the 30 kDa mature form is the main one detected.

We previously reported that exposure to an environmentally relevant OC mixture disrupts cAMP-induced steroidogenesis in two Leydig cell lines, MA-10 and MLTC-1 [18]. In both Leydig cell lines, exposure to the OC mixture causes a dramatic reduction in hormone-induced progesterone (MA-10) and testosterone (MLTC-1) production [18]. In addition, this OC mixture was recently reported to affect various male reproductive parameters across several generations [22,33,34,35]. The disruption in steroidogenesis we previously reported was correlated with a significant decrease in the levels of cAMP-induced STAR mature 30 kDa form while having no effect on *Star* gene transcription [18], suggesting that the OC mixture affects STAR protein activity and/or processing. In the present study, treatment with MG132, an inhibitor of protein degradation, led to an important increase in STAR 30 kDa levels as well as the appearance of the precursor 37 kDa protein, consistent with previous reports [23,24]. High levels of the 30 kDa STAR protein observed in the presence of MG132 were still significantly reduced by the OC mixture in the absence or presence of 8Br-cAMP. These data indicate that the OC mixture and MG132 do not interfere with de novo STAR protein synthesis. These data also support the concept that the OC mixture affects STAR protein processing in Leydig cells. 

There are different possibilities that are not mutually exclusive that can explain the mechanism of OC action. For instance, it is possible that that OC mixture reduces the processing of the 37 kDa form into the mature 30 kDa form. The shorter half-life of the 37 kDa form combined with the normal degradation of the 30 kDa by mitochondrial proteases would explain the reduced level of the 30 kDa STAR protein following OC exposure. In the presence of MG132, degradation of the 37 kDa form by the proteasome is reduced, leading to increased availability of STAR pre-protein for import into the mitochondria and therefore increased 30 kDa form. Another possibility is that the OC mixture accelerates the degradation of the 30 kDa STAR protein inside the mitochondrial matrix via a pathway that is normally inhibited by MG132. This is supported by the fact that the OC appears to act by antagonizing, at least in part, the action of MG132. STAR protein is stabilized and accumulates in the presence of MG132, but this is prevented by the OC mixture. Finally, in a previous study using heterologous COS-7 overexpressing STAR, we reported that exposure to the OC mixture causes a dramatic increase in the 37 kDa form, while the 30 kDa form becomes barely detectable [18]. These data, combined with our present work, support the hypothesis that the OC mixture mainly affects the processing of the 37 kDa precursor STAR protein into the 30 kDa mature form, although other mechanisms cannot be formally excluded. STAR protein processing begins in the mitochondrial intermembrane space and is completed within the matrix before undergoing degradation, which involves several proteases including the LON protease homolog LONP1 [29]. In the absence of the LON protease, STAR turnover is arrested [29]. Although the LON protease could be a target for the OC mixture, we did not observe any change in LONP protein levels in MA-10 Leydig cells exposed to the OC mixture (data not shown). We cannot, however, exclude the possibility that the OC mixture could affect the expression of other proteases involved in STAR processing and/or the activity of any protease involved in STAR processing.

From a functional perspective, the OC mixture and MG132 both reduced the 8Br-cAMP-induced progesterone production in Leydig cells. This reduction was more important when the OC mixture and MG132 were combined. This could be explained in part by the STAR overload response, where increased STAR levels in the mitochondrial matrix cause swelling of the mitochondria, often associated with loss of membrane potential and reduced mitochondrial function [24]. Reduced mitochondrial function could then explain the decrease in progesterone production in the presence of MG132 and/or the OC mixture. Alternatively, reduced progesterone production could also be explained by altered expression and/or activity of proteins other than STAR targeted by the OC mixture.

### 3.2. The OC Mixture Globally Affects Proteins Involved in Multiple Biological Processes in Leydig Cells

Steroidogenesis is a cellular process that consists of a series of reactions involving the action of several enzymes and proteins (reviewed in [4,36]). Dysfunction and/or insufficient function of any of these proteins and enzymes results in disruption of steroidogenesis. In our present work, the fact that 8Br-cAMP-induced steroid hormone synthesis was still repressed by the OC mixture in MA-10 Leydig cells in the presence of MG132 (which affects STAR protein processing), indicates that other proteins in addition to STAR are targeted by the OC mixture. We performed proteomic analysis using 2D-SDS-PAGE followed by LC-MS/MS and identified several proteins affected by the OC mixture. Several of the deregulated proteins are known to be involved in energy metabolism, gene expression, mitochondrial transport, lipid metabolic process, and steroidogenesis, all processes essential for proper Leydig cell function.

Amongst the various proteins identified as potentially affected following exposure to the OC mixture, two are key steroidogenic proteins: CYP11A1 and FDXR. Both proteins were found to be reduced in the LC-MS/MS high throughput screen. CYP11A1 and FDXR are essential for the conversion of cholesterol into pregnenolone once cholesterol has entered the mitochondria and insufficiency or dysfunction of either of these proteins has dramatic impacts on steroid hormone biosynthesis (reviewed in [12,36]). Interestingly, in a previous study, we had identified these two proteins as targets of the OC mixture and validated their downregulation by Western blots using protein-specific antibodies [18]. In addition to these two steroidogenic proteins, we have validated that the levels of three additional proteins from the LC-MS/MS high throughput screen were altered: ACADM, ACOT9, and ACO2. ACADM (medium-chain specific acyl-CoA dehydrogenase) and ACOT9 (acyl-coenzyme A thioesterase 9) are two enzymes involved in lipid metabolism. More specifically, ACADM catalyzes the first step of mitochondrial fatty acid β-oxidation and is known to be present in MA-10 Leydig cells [37], while ACOT9 catalyzes the hydrolysis of acyl-CoAs to the free fatty acid and coenzyme A. The protein ACO2 (aconitase) catalyzes the isomerization of citrate to isocitrate, an essential step of the Krebs cycle. The fact that the levels of the cholesterol transporter STAR ([18] and our current work) and of the two proteins involved in fatty acid β-oxidation (ACADM and ACOT9) were reduced by the OC mixture indicate that lipid transport and metabolism is disrupted. This is reminiscent of impaired lipid metabolism caused by exposure of Leydig cells to another endocrine disruptor, the plasticizer mono-2-ethylhexyl phthalate (MEHP), as revealed by the increase in the number of lipid droplets, especially in hormonally stimulated cells ([38] and our unpublished data). This impairment in lipid metabolism coupled with increased levels of ACO2 (Krebs cycle) indicate that the main metabolic pathways in MA-10 Leydig cells are compromised by the OC mixture.

Although our present work identified several novel proteins, pathways, and biological processes that are affected following exposure of MA-10 Leydig cells to an environmentally relevant OC mixture, there are limitations that need to be considered. One is the importance of validation of the bioinformatics data. Even though results from our Western blots validate the data obtained with the LC-MS/MS approach, confirmation of the level of each protein following exposure to the OC mixture remains essential. Another limitation is the use of an immortalized cell line in culture. Although accepted as the gold standard Leydig cell line model, MA-10 Leydig cells are not identical to primary Leydig cells in culture. However, our work in another Leydig cell line (MLTC-1 cells) [18] showed that the OC mixture had similar effects steroidogenesis. Cells in culture (whether primary cells or a cell line) are being studied outside of their natural local environment, which includes crosstalk with other cell types. Similarly, working with a cell in vitro only provides information on the direct effects on that cell type, which might preclude indirect effects mediated through other organs of the hypothalamo-pituitary-gonadal axis and cell types such as neighbouring Sertoli cells.

In conclusion, we showed that an OC mixture negatively impacts steroidogenesis at the posttranslational level by affecting the processing of the STAR protein. We also showed that the OC mixture alters the levels of other proteins important for Leydig cell function. Although our data provide novel insights and identified several potential targets, additional work is still needed to fully decipher the mechanism of action of the OC mixture on the repression of steroid hormone biosynthesis in Leydig cells.

## 4. Materials and Methods

### 4.1. Chemicals

The cAMP analog 8-bromo-cAMP (8Br-cAMP) was purchased from Sigma-Aldrich Canada (Oakville, ON, Canada). MG132, an inhibitor of proteasome enzymes, was purchased from EMD Millipore (Gibbstown, NJ, USA). The OC mixture was designed to approximate levels of organochlorines found in Arctic ringed seal blubber [39], a main diet constituent of the Inuit population in Northern Québec, Canada. The pure mixture of organochlorines (15 components in total) was diluted in dimethylsulfoxyde (DMSO) purchased from Sigma-Aldrich Canada (Oakville, ON, Canada) as described previously [17,18,22,39] to obtain the proportions listed in Table 1. This organochlorine mixture was used in whole animal studies and found to disrupt reproductive development and function in male rats over several generations [22,33,34,35]. The concentration of organochlorines used in our present work (10 µg/mL) is based on an environmentally relevant dose used in in vivo animal studies. This environmentally relevant dose is reported as 500 µg PCBs/kg body weight plus other persistent organic pollutants (POPs) in lower proportions (3.1–330.3 µg/kg body weight each), totalling >1.5 mg POPs/kg body weight [33,34]. The mixture contains 4.2 ng/mL total polychlorinated biphenyls (PCBs), where PCBs represent about 30% of the mixture [22]. A concentration of 4.2 ng/mL corresponds to total PCBs found in plasma samples from Inuit women of reproductive age from Nunavik (1.0–47.9 ng/mL plasma) and Greenland [40].

### 4.2. Cell Culture

Mouse tumor MA-10 Leydig cells [41] were provided by Dr. Mario Ascoli (University of Iowa, Iowa City, IA, USA). The MA-10 Leydig cell line was originally established from a Leydig cell tumour in a mouse (M5480P) [41] and has since been thoroughly characterized. Treatment of MA-10 cells with luteinizing hormone/human chorionic gonadotropin, forskolin, or cAMP leads to increased steroid hormone production ([41] and reviewed in [42]). The MA-10 Leydig cell line corresponds to the adult population of Leydig cells arrested at the immature stage as characterized by high levels of 5α-reductase [43]. Although MA-10 cells contain *Cyp17a1* mRNA, due to a mutation in the *Cyp17a1* coding sequence they are deficient in CYP17A1 activity and therefore produce mainly progesterone [41]. The MA-10 Leydig cell line is considered an appropriate Leydig cell model to study the molecular mechanisms of endocrine disruptor action in these cells. MA-10 cells were cultured in Dulbecco’s Modified Eagle Medium F12 (DMEM-F12) (Fisher Scientific, Nepean, ON, Canada) supplemented with penicillin and streptomycin and 15% horse serum (Fisher Scientific, Nepean, ON, Canada) and then incubated at 37 °C in 5% CO_2_. For OC treatment, cells were cultured in media containing charcoal-treated serum to remove traces of steroids. MA-10 cells were incubated for a total of 6 h with vehicle (DMSO) or 10 µg/mL of an environmentally relevant mixture of over 15 OCs. This concentration of 10 µg/mL was previously found to be optimal for cell line studies and does not affect mitochondrial integrity and viability of Leydig cells [18]. Two hours after the treatment with OC had begun, cells were stimulated or not with 0.1 mM 8Br-cAMP, with or without 10 µM of MG132 for 4 h.

### 4.3. Protein Purification and Western Blot

MA-10 Leydig cells were seeded in 6-well plates at 500,000 cells per well and treated as described above. Treated cells were rinsed twice with PBS and harvested for total protein extraction. Total proteins were isolated by lysing the cells directly into RIPA buffer (50 mM Tris-HCl (pH 7.5), 0.5% Igepal, 150 mM NaCl, 1 mM EDTA, 1 mM dithiothreitol (DTT), 0.5 mM phenylmethanesulfonylfluoride (PMSF), 10 µg/mL aprotinin, 1 µg/mL each for leupeptin, and pepstatin) for 20 min at 4 °C, followed by one 10-s pulse of sonication and then centrifuged to remove cell debris. Protein concentrations were estimated using standard Bradford assays. Ten or fifteen µg of total proteins were boiled for 2 min in a denaturing loading buffer (20% glycerol, 4% SDS, 100 mM Tris pH 6.8, 0.002% Bromophenol blue, 4% β-Mercaptoethanol), separated by SDS-PAGE, and transferred onto polyvinylidene difluoride (PVDF) membrane (Millipore, Bedford, MA, USA). STAR immunodetection was performed using an avidin-biotin approach according to the manufacturer’s instructions (Vector Laboratories, Inc., Burlington, ON, Canada). For αTUBULIN, an ECL-HRP detection kit (GE Healthcare Life Sciences, Baie d’Urfé, QC, Canada) was used according to the manufacturer’s instructions. For ACADM, ACO2, ACOT9, and GAPDH, the Clarity Western ECL Substrate with an HRP-conjugated secondary anti-rabbit or anti-mouse antibody was used according to the manufacturer’s recommendations (Bio-Rad Laboratories Canada, Mississauga, ON, Canada). Immunodetections were performed using a rabbit anti-STAR antiserum (FL-285, 1:1000 dilution; Santa Cruz Biotechnologies, Santa Cruz, CA, USA), a rabbit anti-ACADM antiserum (1:2000 dilution, Sigma-Aldrich Canada, Oakville, ON, Canada), a rabbit anti-ACO2 antiserum (1:2000 dilution, Sigma-Aldrich Canada, Oakville, ON, Canada), a rabbit anti-ACOT9 antiserum (1:2000 dilution, Sigma-Aldrich Canada, Oakville, ON, Canada), a monoclonal mouse anti-GAPDH antibody (1:5000 dilution, Santa Cruz Biotechnologies, Santa Cruz, USA), and a mouse monoclonal anti-αTUBULIN antibody (1:10000 dilution; Sigma-Aldrich Canada, Oakville, ON, Canada). All experiments were repeated at least four times and produced similar results. Protein levels were quantified using Image Lab (version 6.1.0 build 7, Bio-Rad Laboratories) or Fiji [44] software and normalized with either GAPDH or αTUBULIN levels, which were used as internal loading controls.

### 4.4. Progesterone Quantification

ELISAs for progesterone quantification were performed as recommended by the manufacturer (Cayman Chemical, Ann Arbor, MI, USA) as described previously [17,18,45,46].

### 4.5. Protein Purification and Two-Dimensional SDS-PAGE

MA-10 Leydig cells were seeded in 10 cm petri dishes at 3,000,000 cells/dish and treated as described above. Treated cells were rinsed twice with PBS and harvested for total protein extractions. Total proteins were isolated by lysing the cells directly into 2D buffer (7M urea, 2M thiourea, 4% 3-[(3-Cholamidopropyl)-dimethylammonio-propane-sulfonate (CHAPS), 2% pharmalytes, 40 mM dithiothreitol (DTT)) for 2 h at room temperature, followed by centrifugation to remove cell debris. Protein concentrations were estimated using standard Bradford assays. Eight hundred µg of total proteins in a volume of 250 µL were loaded on immobilized pH gradient strips, pH 3–10, 13 cm (GE Healthcare Life Sciences, Baie d’Urfé, QC, Canada) for isoelectric focusing (IEF). IEF was carried out on an IPGphor II IEF system (GE Healthcare Life Sciences, Baie d’Urfé, QC, Canada). Gels were subjected to 30 V for 12 h, 250 V for 30 min, 500 V for 1 h, 1000 V for 1 h, 3000 V for 1 h, and 8000 V until 16000 V was reached. Gels were first equilibrated using SDS equilibration buffer (urea 6 M, 75 mM Tris-HCl pH 8.8, 3% glycerol, 2% SDS, 1% bromophenol blue) containing 65 mM DTT for 15 min at room temperature and then in SDS equilibration buffer containing iodoacetamide 25 mg/mL for 15 min. Equilibrated gels were then sealed in 3% SDS-PAGE stacking acrylamide gel using 0.5% agarose in standard Tris-glycine electrophoresis buffer. Second dimension 7.5% SDS-PAGE separation acrylamide gel was run at 140 V until the tracking dye had run off the gel.

### 4.6. Gel Fixation, Staining and Analysis

Gels were fixed with fixation buffer (45% methanol and 1% acetic acid) for 2 h, and were then rinsed 3 times with distilled water. Fixed gels were stained using colloidal Coomassie blue solution (10% acetic acid, 0.1 g/mL ammonium sulfate, 1.2 mg/mL Coomassie blue G-250, 0.2% methanol) overnight. Stained gels were rinsed with distilled water until protein spots were visible. Gels were scanned using the Multilmage Light Cabinet (ProteinSimple, Ottawa, ON, Canada), and images were analyzed using ImageMaster 2D Platinum 6.0 software (GE Healthcare Life Sciences, Baie d’Urfé, QC, Canada), which allows for gel matching and spot quantification. Two spots that were different after gel matching and between all conditions were chosen, manually excised, and sent for identification using LC-MS/MS (Proteomics core service, Centre recherche du CHU de Québec-Université Laval, Québec, QC, Canada).

### 4.7. LC-MS/MS and Bioinformatic Data Analysis

LC-MS/MS results were analyzed using Scaffold software (Proteome Software Inc. Portland, OR, USA). Filters used were protein threshold 99%, minimum unique peptide 2, and peptide threshold 95%. The proteins present in the two samples (Sample 1 and Sample 2) were analyzed using the String software, accessed on 26 November 2021 [47] (string-db.org/), while the OriginPro Version 2021 software, accessed on 26 November 2021 (www.originlab.com) (OriginLab Corporation, Northampton, MA, USA) was used to generate the Venn diagram and the heatmap for differentially expressed proteins.

### 4.8. Statistical Analysis

For all single comparisons between two experimental groups, paired Student’s *t* tests were performed. To identify significant differences between multiple groups, statistical analyses were carried out using a nonparametric one-way ANOVA on ranks via Kruskal–Wallis test followed by a Tukey HSD test to detect differences between pairs. For all statistical analyses, *p* < 0.05 was considered significant. All statistical analyses were performed using GraphPad Prism software (La Jolla, CA, USA). For the String analysis, the Strength represents the ratio between the number of proteins in our sample network that are annotated with a term and the total number of proteins annotated with this term in a random network of the same size. The False Discovery Rate (FDR) refers to the significance of the enrichment and shown as *p*-values corrected for multiple testing within each category using the Benjamini–Hochberg procedure [48].

## Figures and Tables

**Table 1:** Composition of the organochlorine mixture [22] used in this study.

Compound	CAS no.	% Weight
PCB mixture *		32.6
Technical chlordane	57-74-9	21.3
P,P’-DDE	72-55-9	19.3
P,P’-DDT	50-29-3	6.79
Technical toxaphene	8001-35-2	6.54
α-HCH	319-84-6	6.17
Aldrin	309-00-2	2.52
Dieldrin	60-57-1	2.09
1,2,4,5-Tetrachlorobenzene	95-94-3	0.86
P,P’-DDD	72-54-8	0.49
β-HCH	319-85-7	0.46
Hexachlorobenzene	118-74-1	0.35
Mirex	2385-85-5	0.23
γ-HCH	58-89-9	0.20
Pentachlorobenzene	608-93-5	0.18

* Polychlorinated biphenyl (PCB) Mix: Aroclor 1260 (58.9%), Aroclor 1254 (39.3%), 2,4,40-triclorobiphenyl (1.0%), 2,20,4,40-tetrachlorobiphenyl (0.8%), 3,4,5,30,40-PCB (PCB 126; 0.02%), and 3,30,4,40-tetrachlorobiphenyl (0.004%).

**Table 2:** Biological processes associated with proteins present in Sample 1.

GO Term ID	Term Description-Biological Process	Observed Gene Count	Background Gene Count	Strength	FDR	Matching Proteins in Your Network (Labels)
GO:0009987	Cellular process	33	13330	0.18	0.0196	Mcm7,Letm1,Qars,Canx,Ncbp3,Tars,Aco2,Hsp90ab1,Thoc1,Aldh18a1,Atp5b,Xrcc5,Ncl,Vcp,Dhx15,Gas6,Vps35,Cog4,Acsbg1,Appl1,Hook3,Srsf4,Actg1,Snrnp70,Gspt1,Hsp90aa1,Cttn,Uba2,Tnpo1,Mcm5,Rangap1,Eif4b,Slc3a2
GO:0044238	Primary metabolic process	23	6369	0.34	0.0145	Mcm7,Qars,Ncbp3,Tars,Aco2,Hsp90ab1,Thoc1,Aldh18a1,Atp5b,Xrcc5,Vcp,Dhx15,Gas6,Vps35,Acsbg1,Srsf4,Snrnp70,Gspt1,Hsp90aa1,Uba2,Mcm5,Eif4b,Slc3a2
GO:0071704	Organic substance metabolic process	23	6824	0.31	0.0271	Mcm7,Qars,Ncbp3,Tars,Aco2,Hsp90ab1,Thoc1,Aldh18a1,Atp5b,Xrcc5,Vcp,Dhx15,Gas6,Vps35,Acsbg1,Srsf4,Snrnp70,Gspt1,Hsp90aa1,Uba2,Mcm5,Eif4b,Slc3a2
GO:0044237	Cellular metabolic process	22	6445	0.32	0.0350	Mcm7,Qars,Ncbp3,Tars,Aco2,Hsp90ab1,Thoc1,Aldh18a1,Atp5b,Xrcc5,Vcp,Dhx15,Gas6,Vps35,Acsbg1,Srsf4,Snrnp70,Gspt1,Hsp90aa1,Uba2,Mcm5,Eif4b
GO:0051179	Localization	18	4646	0.38	0.0445	Letm1,Canx,Ncbp3,Thoc1,Atp5b,Ncl,Vcp,Gas6,Vps35,Cog4,Appl1,Hook3,Actg1,Hsp90aa1,Cttn,Tnpo1,Rangap1,Slc3a2
GO:0006810	Transport	17	3382	0.49	0.0116	Letm1,Canx,Ncbp3,Thoc1,Atp5b,Ncl,Vcp,Gas6,Vps35,Cog4,Appl1,Hook3,Actg1,Hsp90aa1,Cttn,Tnpo1,Slc3a2
GO:0046483	Heterocycle metabolic process	16	2347	0.62	0.0044	Mcm7,Qars,Ncbp3,Tars,Hsp90ab1,Thoc1,Aldh18a1,Atp5b,Xrcc5,Vcp,Dhx15,Srsf4,Snrnp70,Gspt1,Hsp90aa1,Mcm5
GO:1901360	Organic cyclic compound metabolic process	16	2614	0.57	0.0048	Mcm7,Qars,Ncbp3,Tars,Hsp90ab1,Thoc1,Aldh18a1,Atp5b,Xrcc5,Vcp,Dhx15,Srsf4,Snrnp70,Gspt1,Hsp90aa1,Mcm5
GO:0034641	Cellular nitrogen compound metabolic process	16	2805	0.54	0.0070	Mcm7,Qars,Ncbp3,Tars,Hsp90ab1,Thoc1,Atp5b,Xrcc5,Vcp,Dhx15,Srsf4,Snrnp70,Gspt1,Hsp90aa1,Mcm5,Eif4b
GO:0006139	Nucleobase-containing compound metabolic process	15	2205	0.62	0.0044	Mcm7,Qars,Ncbp3,Tars,Hsp90ab1,Thoc1,Atp5b,Xrcc5,Vcp,Dhx15,Srsf4,Snrnp70,Gspt1,Hsp90aa1,Mcm5
GO:0010033	Response to organic substance	15	2742	0.53	0.0141	Mcm7,Canx,Hsp90ab1,Atp5b,Xrcc5,Ncl,Vcp,Dhx15,Gas6,Acsbg1,Appl1,Actg1,Hsp90aa1,Rangap1,Slc3a2
GO:0090304	Nucleic acid metabolic process	14	1777	0.68	0.0044	Mcm7,Qars,Ncbp3,Tars,Hsp90ab1,Thoc1,Xrcc5,Vcp,Dhx15,Srsf4,Snrnp70,Gspt1,Hsp90aa1,Mcm5
GO:0051641	Cellular localization	14	2115	0.61	0.0070	Letm1,Canx,Thoc1,Atp5b,Vcp,Gas6,Vps35,Appl1,Hook3,Actg1,Hsp90aa1,Cttn,Tnpo1,Rangap1
GO:0022607	Cellular component assembly	13	1983	0.6	0.0130	Mcm7,Letm1,Hsp90ab1,Xrcc5,Vcp,Gas6,Vps35,Cog4,Actg1,Hsp90aa1,Cttn,Mcm5,Eif4b
GO:0044249	Cellular biosynthetic process	13	2025	0.59	0.0140	Mcm7,Qars,Tars,Hsp90ab1,Thoc1,Aldh18a1,Atp5b,Vcp,Acsbg1,Gspt1,Hsp90aa1,Mcm5,Eif4b
GO:1901576	Organic substance biosynthetic process	13	2121	0.57	0.0164	Mcm7,Qars,Tars,Hsp90ab1,Thoc1,Aldh18a1,Atp5b,Vcp,Acsbg1,Gspt1,Hsp90aa1,Mcm5,Eif4b
GO:0033036	Macromolecule localization	13	2198	0.56	0.0194	Ncbp3,Thoc1,Vcp,Gas6,Vps35,Cog4,Appl1,Hook3,Actg1,Hsp90aa1,Cttn,Tnpo1,Rangap1
GO:0051649	Establishment of localization in cell	12	1518	0.68	0.0070	Letm1,Canx,Thoc1,Atp5b,Vcp,Vps35,Appl1,Hook3,Actg1,Hsp90aa1,Cttn,Tnpo1
GO:0008104	Protein localization	12	1890	0.59	0.0194	Thoc1,Vcp,Gas6,Vps35,Cog4,Appl1,Hook3,Actg1,Hsp90aa1,Cttn,Tnpo1,Rangap1
GO:0043933	Protein-containing complex subunit organization	11	1164	0.76	0.0057	Mcm7,Letm1,Hsp90ab1,Xrcc5,Vcp,Cog4,Gspt1,Hsp90aa1,Cttn,Mcm5,Eif4b
GO:0071705	Nitrogen compound transport	11	1534	0.64	0.0194	Ncbp3,Thoc1,Vcp,Vps35,Cog4,Appl1,Hook3,Hsp90aa1,Cttn,Tnpo1,Slc3a2
GO:0010467	Gene expression	11	1743	0.59	0.0350	Qars,Ncbp3,Tars,Thoc1,Dhx15,Gas6,Vps35,Srsf4,Snrnp70,Gspt1,Eif4b
GO:0071702	Organic substance transport	11	1836	0.56	0.0445	Ncbp3,Thoc1,Vcp,Vps35,Cog4,Appl1,Hook3,Hsp90aa1,Cttn,Tnpo1,Slc3a2
GO:0016192	Vesicle-mediated transport	10	1130	0.73	0.0140	Canx,Atp5b,Ncl,Vcp,Gas6,Vps35,Cog4,Hook3,Actg1,Cttn
GO:0034645	Cellular macromolecule biosynthetic process	10	1233	0.7	0.0188	Mcm7,Qars,Tars,Hsp90ab1,Thoc1,Vcp,Gspt1,Hsp90aa1,Mcm5,Eif4b
GO:0034613	Cellular protein localization	10	1392	0.64	0.0312	Thoc1,Vcp,Gas6,Vps35,Appl1,Hook3,Hsp90aa1,Cttn,Tnpo1,Rangap1
GO:0065003	Protein-containing complex assembly	9	1025	0.73	0.0196	Mcm7,Letm1,Hsp90ab1,Xrcc5,Cog4,Hsp90aa1,Cttn,Mcm5,Eif4b
GO:0046907	Intracellular transport	9	1166	0.67	0.0390	Thoc1,Atp5b,Vcp,Vps35,Appl1,Hook3,Hsp90aa1,Cttn,Tnpo1
GO:0015031	Protein transport	9	1204	0.66	0.0430	Thoc1,Vcp,Vps35,Cog4,Appl1,Hook3,Hsp90aa1,Cttn,Tnpo1
GO:0034622	Cellular protein-containing complex assembly	8	683	0.86	0.0141	Mcm7,Hsp90ab1,Xrcc5,Cog4,Hsp90aa1,Cttn,Mcm5,Eif4b
GO:1903827	Regulation of cellular protein localization	7	576	0.87	0.0216	Hsp90ab1,Gas6,Vps35,Appl1,Hsp90aa1,Ankrd13a,Rangap1
GO:0006259	DNA metabolic process	7	638	0.83	0.0350	Mcm7,Hsp90ab1,Thoc1,Xrcc5,Vcp,Hsp90aa1,Mcm5
GO:0071345	Cellular response to cytokine stimulus	7	698	0.79	0.0455	Hsp90ab1,Atp5b,Xrcc5,Ncl,Gas6,Appl1,Actg1
GO:0046822	Regulation of nucleocytoplasmic transport	4	129	1.28	0.0328	Hsp90ab1,Gas6,Hsp90aa1,Rangap1
GO:0009651	Response to salt stress	3	31	1.77	0.0194	Hsp90ab1,Xrcc5,Hsp90aa1
GO:0034605	Cellular response to heat	3	57	1.51	0.0466	Hsp90ab1,Vcp,Hsp90aa1
GO:1905323	Telomerase holoenzyme complex assembly	2	3	2.61	0.0194	Hsp90ab1,Hsp90aa1
GO:0006267	Pre-replicative complex assembly involved in nuclear cell cycle DNA replication	2	7	2.24	0.0415	Mcm7,Mcm5
GO:0019062	Virion attachment to host cell	2	7	2.24	0.0415	Hsp90ab1,Gas6

FDR = false discovery rate. Sample 1 correspond to the spots identified by white circles in Figure 3.

**Table 3:** Proteins from Sample 1 present in the different categories from Figure 4B.

A	B	C	D	E	F	G	H	I	J	Other
ACSBG1	THOC1	CTTN	TNPO1	EIF4B	ANKRD13A	DHX15	ACO2	SRSF4	HSP90AB1	HSP90AA1
COG4		GSPT1		HOOK3		TARS	MCM7		HSP90AA1	HNRNPR
		ACTG1		VPS35		MCM5				
		RANGAP1		NCBP3		DHX32				
		NCL		SLC3A2		UBA2				
		VCP		APPL1		ALDH18A1				
		XRCC5		CANX		QARS				
		GAS6		LETM1		SNRNP70				
		ATP5F1B								

The different letters correspond to the category identified by that same letter in Figure 4B.

**Table 4:** Main function of proteins identified in Sample 1 and modulated by the OC mixture (Figure 4C).

Upregulated by 8Br-cAMP and Downregulated by the OC Mixture:	
RANGAP1:	Ran GTPase-activating protein 1. Activation of GTPase activity
XRCC5:	X-ray repair cross-complementing protein 5. Single-stranded DNA-dependent ATP-dependent helicase that plays a key role in DNA non-homologous end joining (NHEJ) by recruiting DNA-PK to DNA
Upregulated by 8Br-cAMP and Upregulated by the OC Mixture:	
VCP:	Transitional endoplasmic reticulum ATPase. Involved in DNA damage response, apoptosis, ER stress, autophagy.
ACO2:	Aconitate hydratase. Catalyzes the isomerization of citrate to isocitrate via cis-aconitate. TCA/Krebs cycle. Energy metabolism.
GAS6:	Growth arrest-specific protein 6. Ligand for tyrosine-protein kinase receptors AXL, TYRO3, and MER, whose signaling is implicated in cell growth and survival, cell adhesion, and cell migration.
ATP5F1B:	ATP synthase subunit beta. Component of the mitochondrial membrane ATP synthase.
ACTG1:	Actin.
GSPT1:	Eukaryotic peptide chain release factor GTP-binding subunit ERF3A. Involved in translation termination, cell growth.
NCL:	Nucleolin. The major nucleolar protein of growing eukaryotic cells. It induces chromatin decondensation by binding to histone H1.
CTTN:	Src substrate cortactin. Contributes to the organization of the actin cytoskeleton and cell shape.
Downregulated by the OC Mixture Independent of 8Br-cAMP:	
MCM7:	DNA replication licensing factor MCM7. Component of the MCM2-7 complex (MCM complex).
ACSBG1:	Long-chain-fatty-acid--CoA ligase. Catalyzes the conversion of fatty acids such as long-chain and very long-chain fatty acids to their active form acyl-CoAs for both synthesis of cellular lipids and degradation via beta-oxidation.
COG4:	Conserved oligomeric Golgi complex subunit 4. Required for normal Golgi function.
SRSF4:	Serine/arginine-rich splicing factor 4. Plays a role in alternative splice site selection during pre-mRNA splicing.
THOC1:	THO complex subunit 1. Required for efficient export of polyadenylated RNA. Participates in an apoptotic pathway. Essential for early embryonic development. Required for normal gene expression during postnatal testis development.

**Table 5:** Biological processes associated with proteins present in Sample 2.

**GO Term ID**	Term Description-Biological Process	Observed Gene Count	Background Gene Count	Strength	FDR	Matching Proteins in Your Network (Labels)
GO:0009987	Cellular process	63	13330	0.2	1.37 × 10^−7^	Rpa1,Tubb5,Cs,Ap2m1,Pipox,Fdxr,Pafah1b1,Mrps30,Psmc6,Trmu,Hsd17b4,Fen1,Acot9,Prim1,Atp5a1,Eif4a3,Acadl,Bcs1l,Fam98b,Ppid,Slc25a24,Psip1,Paics,Aldh2,Agk,Uqcrc2,Fbxo22,Cyp11a1,Aldh5a1,Pak1ip1,Eef1a1,Ndufv1,Hpse,Ephx1,Rbmxl1,Pcbp1,Eif2s3x,Acox1,Celf1,Actg1,Acadm,Ppox,Acot7,Prpf38a,Eno1,Pgd,Rcc1,Aldoa,Mat2a,Adh7,Hmgcs2,Ssb,Eef1g,Tial1,Idh2,Hnrnpc,Hadhb,Sept7,Cth,Sap30bp,Tbl2,Serpinh1,Micu1
GO:0008152	Metabolic process	53	7331	0.38	1.06 × 10^−10^	Rpa1,Cs,Ap2m1,Pipox,Fdxr,Pafah1b1,Mrps30,Psmc6,Trmu,Hsd17b4,Fen1,Acot9,Prim1,Atp5a1,Eif4a3,Acadl,Fam98b,Ppid,Psip1,Paics,Aldh2,Agk,Uqcrc2,Fbxo22,Cyp11a1,Gmppa,Aldh5a1,Eef1a1,Ndufv1,Hpse,Ephx1,Rbmxl1,Pcbp1,Eif2s3x,Acox1,Celf1,Acadm,Ppox,Acot7,Prpf38a,Eno1,Pgd,Aldoa,Mat2a,Adh7,Hmgcs2,Ssb,Eef1g,Idh2,Hnrnpc,Hadhb,Cth,Serpinh1
GO:0071704	Organic substance metabolic process	51	6824	0.4	1.06 × 10^−10^	Rpa1,Cs,Ap2m1,Pipox,Fdxr,Pafah1b1,Mrps30,Psmc6,Trmu,Hsd17b4,Fen1,Acot9,Prim1,Atp5a1,Eif4a3,Acadl,Fam98b,Ppid,Psip1,Paics,Aldh2,Agk,Uqcrc2,Fbxo22,Cyp11a1,Aldh5a1,Eef1a1,Hpse,Ephx1,Rbmxl1,Pcbp1,Eif2s3x,Acox1,Celf1,Acadm,Ppox,Acot7,Prpf38a,Eno1,Pgd,Aldoa,Mat2a,Adh7,Hmgcs2,Ssb,Eef1g,Idh2,Hnrnpc,Hadhb,Cth,Serpinh1
GO:0044237	Cellular metabolic process	50	6445	0.41	1.06 × 10^−10^	Rpa1,Cs,Ap2m1,Pipox,Fdxr,Mrps30,Psmc6,Trmu,Hsd17b4,Fen1,Acot9,Prim1,Atp5a1,Eif4a3,Acadl,Fam98b,Ppid,Psip1,Paics,Aldh2,Agk,Uqcrc2,Fbxo22,Cyp11a1,Aldh5a1,Eef1a1,Ndufv1,Hpse,Ephx1,Rbmxl1,Pcbp1,Eif2s3x,Acox1,Celf1,Acadm,Ppox,Acot7,Prpf38a,Eno1,Pgd,Aldoa,Mat2a,Adh7,Hmgcs2,Ssb,Eef1g,Idh2,Hnrnpc,Hadhb,Cth
GO:0044238	Primary metabolic process	47	6369	0.39	6.80 × 10^−9^	Rpa1,Cs,Pipox,Fdxr,Pafah1b1,Mrps30,Psmc6,Trmu,Hsd17b4,Fen1,Acot9,Prim1,Atp5a1,Eif4a3,Acadl,Fam98b,Ppid,Psip1,Paics,Agk,Uqcrc2,Fbxo22,Cyp11a1,Aldh5a1,Eef1a1,Hpse,Ephx1,Rbmxl1,Pcbp1,Eif2s3x,Acox1,Celf1,Acadm,Acot7,Prpf38a,Eno1,Pgd,Aldoa,Adh7,Hmgcs2,Ssb,Eef1g,Idh2,Hnrnpc,Hadhb,Cth,Serpinh1
GO:0006807	Nitrogen compound metabolic process	40	5878	0.36	7.55 × 10^−6^	Rpa1,Cs,Pipox,Mrps30,Psmc6,Trmu,Hsd17b4,Fen1,Acot9,Prim1,Atp5a1,Eif4a3,Acadl,Fam98b,Ppid,Psip1,Paics,Agk,Uqcrc2,Fbxo22,Aldh5a1,Eef1a1,Hpse,Rbmxl1,Pcbp1,Eif2s3x,Celf1,Acadm,Ppox,Acot7,Prpf38a,Eno1,Aldoa,Hmgcs2,Ssb,Eef1g,Idh2,Hnrnpc,Cth,Serpinh1
GO:0034641	Cellular nitrogen compound metabolic process	32	2805	0.58	7.04 × 10^−9^	Rpa1,Cs,Pipox,Mrps30,Trmu,Hsd17b4,Fen1,Acot9,Prim1,Atp5a1,Eif4a3,Acadl,Psip1,Paics,Agk,Eef1a1,Rbmxl1,Pcbp1,Eif2s3x,Celf1,Acadm,Ppox,Acot7,Prpf38a,Eno1,Aldoa,Hmgcs2,Ssb,Eef1g,Idh2,Hnrnpc,Cth
GO:1901564	Organonitrogen compound metabolic process	29	4475	0.34	4.1 × 10^−3^	Cs,Pipox,Mrps30,Psmc6,Hsd17b4,Acot9,Atp5a1,Acadl,Fam98b,Ppid,Paics,Agk,Uqcrc2,Fbxo22,Aldh5a1,Eef1a1,Hpse,Rbmxl1,Eif2s3x,Acadm,Ppox,Acot7,Eno1,Aldoa,Hmgcs2,Eef1g,Idh2,Cth,Serpinh1
GO:1901360	Organic cyclic compound metabolic process	27	2614	0.54	2.61 × 10^−6^	Rpa1,Cs,Pipox,Fdxr,Trmu,Hsd17b4,Fen1,Acot9,Prim1,Atp5a1,Eif4a3,Psip1,Paics,Cyp11a1,Ephx1,Rbmxl1,Pcbp1,Celf1,Ppox,Acot7,Prpf38a,Eno1,Aldoa,Hmgcs2,Ssb,Idh2,Hnrnpc
GO:0009058	Biosynthetic process	25	2176	0.58	1.90 × 10^−6^	Rpa1,Fdxr,Mrps30,Fen1,Prim1,Atp5a1,Paics,Agk,Cyp11a1,Gmppa,Eef1a1,Ephx1,Rbmxl1,Eif2s3x,Acadm,Ppox,Acot7,Pgd,Aldoa,Mat2a,Hmgcs2,Eef1g,Idh2,Cth,Serpinh1
GO:0046483	Heterocycle metabolic process	25	2347	0.55	6.8 × 10^−6^	Rpa1,Cs,Pipox,Trmu,Hsd17b4,Fen1,Acot9,Prim1,Atp5a1,Eif4a3,Psip1,Paics,Ephx1,Rbmxl1,Pcbp1,Celf1,Ppox,Acot7,Prpf38a,Eno1,Aldoa,Hmgcs2,Ssb,Idh2,Hnrnpc
GO:0006725	Cellular aromatic compound metabolic process	25	2412	0.54	1 × 10^−5^	Rpa1,Cs,Pipox,Trmu,Hsd17b4,Fen1,Acot9,Prim1,Atp5a1,Eif4a3,Psip1,Paics,Ephx1,Rbmxl1,Pcbp1,Celf1,Ppox,Acot7,Prpf38a,Eno1,Aldoa,Hmgcs2,Ssb,Idh2,Hnrnpc
GO:0044281	Small molecule metabolic process	24	1450	0.74	7.04 × 10^−9^	Cs,Pipox,Fdxr,Hsd17b4,Acot9,Atp5a1,Acadl,Paics,Aldh2,Cyp11a1,Aldh5a1,Ephx1,Acox1,Acadm,Acot7,Eno1,Pgd,Aldoa,Mat2a,Adh7,Hmgcs2,Idh2,Hadhb,Cth
GO:1901576	Organic substance biosynthetic process	23	2121	0.56	1.52 × 10^−5^	Rpa1,Fdxr,Mrps30,Fen1,Prim1,Atp5a1,Paics,Agk,Cyp11a1,Eef1a1,Ephx1,Rbmxl1,Eif2s3x,Acadm,Ppox,Acot7,Pgd,Aldoa,Mat2a,Hmgcs2,Eef1g,Idh2,Cth
GO:0006139	Nucleobase-containing compound metabolic process	23	2205	0.54	2.84 × 10^−5^	Rpa1,Cs,Pipox,Trmu,Hsd17b4,Fen1,Acot9,Prim1,Atp5a1,Eif4a3,Psip1,Paics,Rbmxl1,Pcbp1,Celf1,Acot7,Prpf38a,Eno1,Aldoa,Hmgcs2,Ssb,Idh2,Hnrnpc
GO:0044249	Cellular biosynthetic process	21	2025	0.54	1.2 × 10^−4^	Rpa1,Fdxr,Mrps30,Fen1,Prim1,Atp5a1,Paics,Agk,Cyp11a1,Eef1a1,Rbmxl1,Eif2s3x,Acadm,Ppox,Acot7,Aldoa,Mat2a,Hmgcs2,Eef1g,Idh2,Cth
GO:0009056	Catabolic process	20	1680	0.6	3.22 × 10^−5^	Pipox,Pafah1b1,Psmc6,Hsd17b4,Fen1,Eif4a3,Acadl,Aldh2,Aldh5a1,Hpse,Ephx1,Acox1,Acadm,Acot7,Eno1,Pgd,Aldoa,Adh7,Ssb,Hadhb
GO:1901575	Organic substance catabolic process	19	1425	0.65	1.52 × 10^−5^	Pipox,Pafah1b1,Psmc6,Hsd17b4,Fen1,Eif4a3,Acadl,Aldh2,Aldh5a1,Hpse,Acox1,Acadm,Acot7,Eno1,Pgd,Aldoa,Adh7,Ssb,Hadhb
GO:0055114	Oxidation-reduction process	18	917	0.82	3.49 × 10^−7^	Cs,Pipox,Fdxr,Hsd17b4,Acadl,Aldh2,Uqcrc2,Cyp11a1,Aldh5a1,Ndufv1,Acox1,Acadm,Ppox,Eno1,Pgd,Adh7,Idh2,Hadhb
GO:0044085	Cellular component biogenesis	18	2201	0.44	1.69 × 10^−2^	Tubb5,Ap2m1,Pafah1b1,Eif4a3,Bcs1l,Ppid,Psip1,Pak1ip1,Rbmxl1,Eif2s3x,Celf1,Actg1,Rcc1,Aldoa,Mat2a,Sept7,Cth,Micu1
GO:0019752	Carboxylic acid metabolic process	16	754	0.85	1.18 × 10^−6^	Cs,Pipox,Hsd17b4,Acadl,Aldh5a1,Ephx1,Acox1,Acadm,Acot7,Eno1,Pgd,Aldoa,Adh7,Idh2,Hadhb,Cth
GO:0022607	Cellular component assembly	16	1983	0.43	4.86 × 10^−2^	Tubb5,Ap2m1,Pafah1b1,Bcs1l,Ppid,Psip1,Rbmxl1,Eif2s3x,Celf1,Actg1,Rcc1,Aldoa,Mat2a,Sept7,Cth,Micu1
GO:0044271	Cellular nitrogen compound biosynthetic process	15	1156	0.64	5.3 × 10^−4^	Rpa1,Mrps30,Prim1,Atp5a1,Paics,Agk,Eef1a1,Rbmxl1,Eif2s3x,Acadm,Ppox,Acot7,Aldoa,Eef1g,Idh2
GO:0044248	Cellular catabolic process	15	1445	0.54	6.3 × 10^−3^	Pipox,Psmc6,Hsd17b4,Fen1,Eif4a3,Acadl,Aldh5a1,Hpse,Ephx1,Acox1,Acadm,Acot7,Pgd,Ssb,Hadhb
GO:0010467	Gene expression	15	1743	0.46	4.06 × 10^−2^	Mrps30,Trmu,Eif4a3,Psip1,Uqcrc2,Eef1a1,Rbmxl1,Pcbp1,Eif2s3x,Celf1,Prpf38a,Ssb,Eef1g,Hnrnpc,Serpinh1
GO:0006629	Lipid metabolic process	14	1032	0.66	7.3 × 10^−4^	Fdxr,Pafah1b1,Hsd17b4,Atp5a1,Acadl,Agk,Cyp11a1,Ephx1,Acox1,Acadm,Acot7,Adh7,Hmgcs2,Hadhb
GO:0032787	Monocarboxylic acid metabolic process	13	461	0.97	1.82 × 10^−6^	Hsd17b4,Acadl,Aldh5a1,Ephx1,Acox1,Acadm,Acot7,Eno1,Pgd,Aldoa,Adh7,Idh2,Hadhb
GO:1901566	Organonitrogen compound biosynthetic process	13	1096	0.6	6.3 × 10^−3^	Mrps30,Atp5a1,Paics,Agk,Eef1a1,Eif2s3x,Acadm,Ppox,Acot7,Aldoa,Eef1g,Idh2,Cth
GO:0044282	Small molecule catabolic process	12	318	1.1	4.65 × 10^−7^	Pipox,Hsd17b4,Acadl,Aldh2,Aldh5a1,Acox1,Acadm,Acot7,Eno1,Pgd,Adh7,Hadhb
GO:0043603	Cellular amide metabolic process	12	689	0.76	3.7 × 10^−4^	Cs,Pipox,Mrps30,Hsd17b4,Acot9,Agk,Eef1a1,Eif2s3x,Acot7,Hmgcs2,Eef1g,Cth
GO:0019637	Organophosphate metabolic process	12	724	0.74	6 × 10^−4^	Cs,Pipox,Hsd17b4,Acot9,Atp5a1,Paics,Acot7,Eno1,Pgd,Aldoa,Hmgcs2,Idh2
GO:1901135	Carbohydrate derivative metabolic process	12	814	0.69	1.8 × 10^−3^	Cs,Pipox,Hsd17b4,Acot9,Atp5a1,Paics,Hpse,Acot7,Eno1,Pgd,Aldoa,Hmgcs2
GO:0009117	Nucleotide metabolic process	11	361	1.01	1.03 × 10^−5^	Cs,Pipox,Hsd17b4,Acot9,Atp5a1,Paics,Acot7,Eno1,Aldoa,Hmgcs2,Idh2
GO:1901362	Organic cyclic compound biosynthetic process	11	883	0.62	1.7 × 10^−2^	Rpa1,Prim1,Atp5a1,Paics,Cyp11a1,Rbmxl1,Ppox,Acot7,Aldoa,Hmgcs2,Idh2
GO:0009150	Purine ribonucleotide metabolic process	10	265	1.1	7.91 × 10^−6^	Cs,Pipox,Hsd17b4,Acot9,Atp5a1,Paics,Acot7,Eno1,Aldoa,Hmgcs2
GO:0006091	Generation of precursor metabolites and energy	10	319	1.02	2.73 × 10^−5^	Cs,Atp5a1,Ndufv1,Acox1,Acadm,Eno1,Pgd,Aldoa,Adh7,Idh2
GO:0044255	Cellular lipid metabolic process	10	797	0.62	3.38 × 10^−2^	Hsd17b4,Acadl,Agk,Ephx1,Acox1,Acadm,Acot7,Adh7,Hmgcs2,Hadhb
GO:0046395	Carboxylic acid catabolic process	9	206	1.16	1.14 × 10^−5^	Pipox,Hsd17b4,Acadl,Aldh5a1,Acox1,Acadm,Acot7,Pgd,Hadhb
GO:0072329	Monocarboxylic acid catabolic process	8	99	1.43	1.67 × 10^−6^	Hsd17b4,Acadl,Aldh5a1,Acox1,Acadm,Acot7,Pgd,Hadhb
GO:0006790	Sulfur compound metabolic process	8	280	0.98	8 × 10^−4^	Cs,Pipox,Hsd17b4,Acot9,Acot7,Mat2a,Hmgcs2,Cth
GO:0006631	Fatty acid metabolic process	8	310	0.94	1.6 × 10^−3^	Hsd17b4,Acadl,Ephx1,Acox1,Acadm,Acot7,Adh7,Hadhb
GO:0016071	mRNA metabolic process	8	516	0.71	3.99 × 10^−2^	Eif4a3,Psip1,Rbmxl1,Pcbp1,Celf1,Prpf38a,Ssb,Hnrnpc
GO:0030258	Lipid modification	7	212	1.04	1.4 × 10^−3^	Hsd17b4,Acadl,Agk,Acox1,Acadm,Adh7,Hadhb
GO:0016042	Lipid catabolic process	7	260	0.95	4.8 × 10^−3^	Pafah1b1,Hsd17b4,Acadl,Acox1,Acadm,Acot7,Hadhb
GO:0006066	Alcohol metabolic process	7	292	0.9	8.6 × 10^−3^	Fdxr,Aldh2,Cyp11a1,Ephx1,Adh7,Hmgcs2,Idh2
GO:0006397	mRNA processing	7	399	0.77	4.86 × 10^−2^	Eif4a3,Psip1,Rbmxl1,Pcbp1,Celf1,Prpf38a,Hnrnpc
GO:0019395	Fatty acid oxidation	6	77	1.42	8.83 × 10^−5^	Hsd17b4,Acadl,Acox1,Acadm,Adh7,Hadhb
GO:0009062	Fatty acid catabolic process	6	79	1.4	9.91 × 10^−5^	Hsd17b4,Acadl,Acox1,Acadm,Acot7,Hadhb
GO:0006637	acyl-CoA metabolic process	6	87	1.36	1.6 × 10^−4^	Cs,Pipox,Hsd17b4,Acot9,Acot7,Hmgcs2
GO:0006839	Mitochondrial transport	6	184	1.04	6.6 × 10^−3^	Atp5a1,Bcs1l,Slc25a24,Agk,Uqcrc2,Micu1
GO:0000398	mRNA splicing, via spliceosome	6	191	1.02	8 × 10^−3^	Eif4a3,Psip1,Rbmxl1,Celf1,Prpf38a,Hnrnpc
GO:0006635	Fatty acid beta-oxidation	5	54	1.49	3.6 × 10^−4^	Hsd17b4,Acadl,Acox1,Acadm,Hadhb
GO:0006376	mRNA splice site selection	3	27	1.57	2.54 × 10^−2^	Psip1,Rbmxl1,Celf1
GO:0006084	acetyl-CoA metabolic process	3	32	1.5	3.94 × 10^−2^	Cs,Pipox,Hmgcs2
GO:0006739	NADP metabolic process	3	32	1.5	3.94 × 10^−2^	Fdxr,Pgd,Idh2
GO:0019254	Carnitine metabolic process, coa-linked	2	3	2.35	2.35 × 10^−2^	Acadl,Acadm
GO:0036112	Medium-chain fatty-acyl-coa metabolic process	2	3	2.35	2.35 × 10^−2^	Hsd17b4,Acot7

FDR = false discovery rate. Sample 2 correspond to the spots identified by yellow circles in Figure 3.

**Table 6:** Proteins from Sample 2 present in the different categories from Figure 5B.

A	B	C	D	E	F	G	H	I
PGD	ADH7	EEF1A1	RBMXL1	AP2M1	BCS1L	TRMU	IDH2	FDXR
	PGK1	CYP11A1	HNRNPC	SSB	ALDOA	HSD17B4		HMGCS2
	ACADM	SEPTIN7	CTH	PAFAH1B1	TIAL1	MICU1		
	PSMC6	AGK	PPID	ALDH5A1	PRPF38A	TBL2		
	CS	CELF1	GMPPA	ALDH2	UQCRC2	FEN1		
	IDH3B	HADHB	SLC25A24	EPHX1	ACADL	FAM98B		
	ACTG1	EIF2S3X	FBXO22	EIF4A3		SAP30BP		
	PAICS	SERPINH1	PCBP1	ACOX				
	ACOT9	PAK1IP1	PIPOX	RPA1				
		TUBB5		PPOX				
		ENO1		ATP5F1A				
		EEF1G		MAT2A				
		MRPS30		HPSE				
		PSIP1		NDUFV1				
		PRIM1		ACOT7				
				RCC1				

The different letters correspond to the category identified by that same letter in Figure 5B.

**Table 7:** Main function of proteins identified in Sample 2 and modulated by the OC mixture (Figure 5C).

UPREGULATED BY 8Br-cAMP	
Downregulated by OC:	
FDXR:	NADPH:adrenodoxin oxidoreductase. Serves as the first electron transfer protein in all the mitochondrial P450 systems, including cholesterol side chain cleavage in all steroidogenic tissues.
TUBB5:	Tubulin beta-5 chain. The major constituent of microtubules.
CYP11A1:	Cholesterol side-chain cleavage enzyme. Catalyzes the side-chain hydroxylation and cleavage of cholesterol to pregnenolone, the precursor of most steroid hormones.
EIF2S3X:	Eukaryotic translation initiation factor 2 subunit 3, X-linked. As a subunit of eukaryotic initiation factor 2 (eIF-2), involved in the early steps of protein synthesis.
EEF1A1:	Elongation factor 1-alpha 1. Promotes the GTP-dependent binding of aminoacyl-tRNA to the A-site of ribosomes during protein biosynthesis.
Upregulated by OC:	
HMGCS2:	Hydroxymethylglutaryl-CoA synthase. Catalyzes the first irreversible step in ketogenesis and cholesterogenesis, condensing acetyl-CoA to acetoacetyl-CoA to form HMG-CoA.
EEF1G:	Elongation factor 1-gamma. Role in translation elongation.
PRIM1:	DNA primase small subunit. Catalytic subunit of the DNA primase complex and component of the DNA polymerase alpha complex (also known as the alpha DNA polymerase-primase complex), which play an essential role in the initiation of DNA synthesis.
CELF1:	CUGBP Elav-like family member 1. RNA-binding protein implicated in the regulation of several post-transcriptional events. Involved in pre-mRNA alternative splicing, mRNA translation, and stability. Mediates exon inclusion and/or exclusion in pre-mRNA that are subject to tissue-specific and developmentally regulated alternative splicing. Increases translation and controls the choice of translation initiation codon of CEBPB mRNA (C/EBPb is an important regulator of several steroidogenic genes in Leydig cells).
MRPS30:	28S ribosomal protein S30. Structural constituent of ribosome.
SEPTIN7:	Septin-7. Filament-forming cytoskeletal GTPase. Required for normal organization of the actin cytoskeleton. Required for normal progress through mitosis. Involved in cytokinesis.
PSIP1:	PC4 and SFRS1-interacting protein. Transcriptional coactivator involved in neuroepithelial stem cell differentiation and neurogenesis. Involved in particular in lens epithelial cell gene regulation and stress responses. May play an important role in lens epithelial to fiber cell terminal differentiation. May play a protective role during stress-induced apoptosis.
ENO1:	Alpha-enolase. Glycolytic enzyme the catalyzes the conversion of 2-phosphoglycerate to phosphoenolpyruvate (precursor for the synthesis of ATP).
HADHB:	Trifunctional enzyme subunit beta. Mitochondrial trifunctional enzyme catalyzes the last three of the four reactions of the mitochondrial beta-oxidation pathway. The mitochondrial beta-oxidation pathway is the major energy-producing process in tissues and is performed through four consecutive reactions, breaking down fatty acids into acetyl-CoA. Among the enzymes involved in this pathway, the trifunctional enzyme exhibits specificity for long-chain fatty acids.
PAK1IP1:	p21-activated protein kinase-interacting protein 1. Negatively regulates the PAK1 kinase. Involved in cell proliferation. May be involved in ribosomal large subunit assembly.
AGK:	Acylglycerol kinase. Lipid kinase that can phosphorylate both monoacylglycerol and diacylglycerol to form lysophosphatidic acid (LPA) and phosphatidic acid (PA), respectively.
SERPINH1:	Serpin H1. Binds specifically to collagen. Could be involved as a chaperone in the biosynthetic pathway of collagen.
DOWNREGULATED BY 8Br-cAMP	
Downregulated by OC:	
ACADM:	Medium-chain specific acyl-CoA dehydrogenase. One of the acyl-CoA dehydrogenases that catalyze the first step of mitochondrial fatty acid beta-oxidation, an aerobic process breaking down fatty acids into acetyl-CoA and allowing the production of energy from fats.
PAICS:	Multifunctional protein ADE2. IMP biosynthesis (SAICAR more specifically).
PSMC6:	26S proteasome regulatory subunit 10B. Component of the 26S proteasome, a multiprotein complex involved in the ATP-dependent degradation of ubiquitinated proteins. This complex plays a key role in the maintenance of protein homeostasis by removing misfolded or damaged proteins, which could impair cellular functions, and by removing proteins whose functions are no longer required.
ACOT9:	Acyl-coenzyme A thioesterase 9. Acyl-CoA thioesterases are a group of enzymes that catalyze the hydrolysis of acyl-CoAs to the free fatty acid and coenzyme A (CoASH), providing the potential to regulate intracellular levels of acyl-CoAs, free fatty acids, and CoASH. Active on long chain acyl-CoAs.
PGD:	6-phosphogluconate dehydrogenase, decarboxylating. Catalyzes the oxidative decarboxylation of 6-phosphogluconate to ribulose 5-phosphate and CO2, with concomitant reduction of NADP to NADPH. Pentose phosphate pathway.
IDH2:	Isocitrate dehydrogenase. Plays a role in intermediary metabolism and energy production.
CS:	Citrate synthase. TCA/Krebs cycle for energy production.
ACTG1:	Actin, cytoplasmic 2. Actins coexist in most cell types as components of the cytoskeleton and as mediators of internal cell motility/contractility.
Upregulated by OC:	
ADH7:	All-trans-retinol dehydrogenase. Catalyzes the NAD-dependent oxidation of all-trans-retinol, alcohol, aldehyde, and omega-hydroxy fatty acids and their derivatives. Therefore, it may participate in retinoid metabolism, fatty acid omega-oxidation, and elimination of cytotoxic aldehydes produced by lipid peroxidation.
IDH3B:	Isocitrate dehydrogenase (NAD) subunit alpha. Catalytic subunit of the enzyme which catalyzes the decarboxylation of isocitrate (ICT) into alpha-ketoglutarate. TCA/Krebs cycle for energy production.
PGK1:	Phosphoglycerate kinase 1. Catalyzes one of the two ATP-producing reactions in the glycolytic pathway via the reversible conversion of 1,3-diphosphoglycerate to 3-phosphoglycerate. In addition to its role as a glycolytic enzyme, it seems that PGK-1 acts as a polymerase alpha cofactor protein (primer recognition protein). May play a role in sperm motility.

## Data Availability

All data generated or analyzed during this study are included in this article.
